# High-throughput, automated quantification of white matter neurons in mild malformation of cortical development in epilepsy

**DOI:** 10.1186/2051-5960-2-72

**Published:** 2014-06-13

**Authors:** Joan YW Liu, Matthew Ellis, Hannah Brooke-Ball, Jane de Tisi, Sofia H Eriksson, Sebastian Brandner, Sanjay M Sisodiya, Maria Thom

**Affiliations:** Department of Clinical and Experimental Epilepsy, UCL Institute of Neurology, Queen Square, WC1N 3BG, London, UK; Division of Neuropathology, National Hospital for Neurology and Neurosurgery, Queen Square, WC1N 3BG, London, UK; Department of Neurodegenerative Disease, UCL Institute of Neurology, Queen Square, WC1N 3BG, London, UK; Epilepsy Society, Chesham Lane, Chalfont St Peter, SL9 0RJ, Bucks, UK

**Keywords:** mMCD, Immunohistochemistry, Quantitation, Stereology, Heterotophic, Neurons

## Abstract

**Introduction:**

In epilepsy, the diagnosis of mild Malformation of Cortical Development type II (mMCD II) predominantly relies on the histopathological assessment of heterotopic neurons in the white matter. The exact diagnostic criteria for mMCD II are still ill-defined, mainly because findings from previous studies were contradictory due to small sample size, and the use of different stains and quantitative systems. Advance in technology leading to the development of whole slide imaging with high-throughput, automated quantitative analysis (WSA) may overcome these differences, and may provide objective, rapid, and reliable quantitation of white matter neurons in epilepsy. This study quantified the density of NeuN immunopositive neurons in the white matter of up to 142 epilepsy and control cases using WSA. Quantitative data from WSA was compared to two other systems, semi-automated quantitation, and the widely accepted method of stereology, to assess the reliability and quality of results from WSA.

**Results:**

All quantitative systems showed a higher density of white matter neurons in epilepsy cases compared to controls (P = 0.002). We found that, in particular, WSA with user-defined region of interest (manual) was superior in terms of larger sampled size, ease of use, time consumption, and accuracy in region selection and cell recognition compared to other methods. Using results from WSA manual, we proposed a threshold value for the classification of mMCD II, where 78% of patients now classified with mMCD II were seizure-free at the second post-operatively follow up.

**Conclusion:**

This study confirms the potential role of WSA in future quantitative diagnostic histology, especially for the histopathological diagnosis of mMCD.

**Electronic supplementary material:**

The online version of this article (doi:10.1186/2051-5960-2-72) contains supplementary material, which is available to authorized users.

## Introduction

According to the Palmini classification, the observation of ‘excess’ heterotrophic neurons in layer I and the white matter are classified as mild forms of Malformation of Cortical Development (mMCD) type I and II respectively [1]. There are presently no other criteria to aid the assessment of mMCD II [2]. Despite many previous studies in this field, the oldest dating back to 1984 [3], it is still uncertain whether the density of WMN is increased in patients with epilepsy, what the threshold for the diagnostic confirmation of mMCD II should be [2], whether the severity of this pathological feature correlates with pre-operative MRI, and represents a clinically relevant, prognostic biomarker, in particular for outcome following epilepsy surgery [4–8]. Findings from previous quantitative studies have been contradictory [3, 9–14], due to the small sample size used (<52 cases), and differing counting methods, equipment, regions of analysis, and cell types studied (Additional file 1: Table S1). Excess density of WMN has also been reported in patients with schizophrenia and autism [15–19], suggesting the possibility that the WMN density may be an indicator of neurodevelopmental disorders beyond epilepsy. Consequently, there is a clinical need for the accurate, rapid evaluation of the numerical threshold of ‘excess’ WMN in epilepsy, and to determine any possible relationships between WMN density and epileptogenesis, pharmacoresistance and surgical outcome.

Recent technological advances in quantitative histology, with systems such as whole slide imaging with high-throughput, automated analysis (WSA), means that it is now possible to more accurately and efficiently quantify histological stains and immunolabelling, which may aid the clinical diagnosis of a wide range of diseases [20–24] and be instrumental in the design of personalised treatment plans which may improve patients’ chances of recovery [25, 26]. In fact, there is an increasing official acceptance of WSA: the United States Food and Drug Administration has approved the WSA of several major companies for the diagnostic evaluation/screening of HER2, PR, ER, Ki67 and/or p53 immunolabelling (Additional file 2: Table S2), underlining the importance and relevance that quantitative histopathology has in personalised medicine. However, before such advanced system is used in routine clinical laboratory, it is important to carefully compare the cost and applicability, and validate the quality of results obtained against traditionally used methods such as semi-automated analysis, and established, gold standard approaches for quantifying the number of neurons in volume of tissue, such as stereology [27].

As such, this study aims to quantify the density of NeuN immunopositive neurons in the white matter of up to 130 samples from patients with epilepsy who have undergone a standardised surgical procedure (anterior temporal lobectomy) for the same underlying pathology (hippocampal sclerosis) and 12 controls using three quantitative methods: (i) WSA, (ii) semi-automated analysis that processes pre-acquired images of the white matter (SA), and (iii) stereology (*n* = 50 cases). This highly homogenous group of epilepsy patients has comprehensive clinical data (Additional file 3: Table S3) to allow us to investigate the clinical relevance of WMN density in epilepsy. Quantification of WMN using the three systems would also allow us to compare the quantitative results, ease of use, time consumption, reliability, and accuracy in the selection of regions and identification of cells.

## Material and methods

This study was approved by the Joint Research Ethics Committee of the National Hospital for Neurology and Neurosurgery and the UCL Institute of Neurology. Patients had given consent to the use of their brain tissue in research, and the tissue was used in accordance with the current UK Human Tissue Authority guidelines. Anterior temporal lobectomy specimens from 130 epilepsy cases and 12 controls (five normal, post mortem controls with no brain pathology, and seven non-epilepsy, surgical controls) were studied. In the epilepsy cases, the resected sample was taken 1 to 1.5 cm from the temporal pole and the sample included the superior, middle, and inferior temporal gyrus with underlying white matter extending to the ventricle. The average age of cases with epilepsy and non-epilepsy surgical controls was 37 years and 39 years respectively. Clinical details of surgical epilepsy and control cases are summarised in Additional file 3: Table S3. Seven to 20 μm-thick, formalin-fixed, paraffin-embedded brain tissue sections from each case were processed and immunolabelled using the marker for mature neurons, Neuronal Nuclei (NeuN, 1:100, Millipore MAB377, UK), and counterstain with haematoxylin (VWR International, UK). The exact section thickness of each case (or the depth of field ‘z’ reading) was later measured using the Zeiss Axio Imager Z2 Motorised microscope with 63× objective lens (average actual thickness ± standard deviation, 17 μm ± 5). NeuN immunopositive cells in the white matter of each sections were counted using (i) WSA: whole slide imaging with high-throughput, automated analytic (Tissue Studio and Developer XD, Germany) with automated and manual ROI selections, and (ii) SA: semi-automated image analytic (ImageProPlus version 6.2, Media Cybernetics, USA), and (iii) three dimensional stereology (Histometrix, Kinetic Imaging, UK).

**Figure 1:**
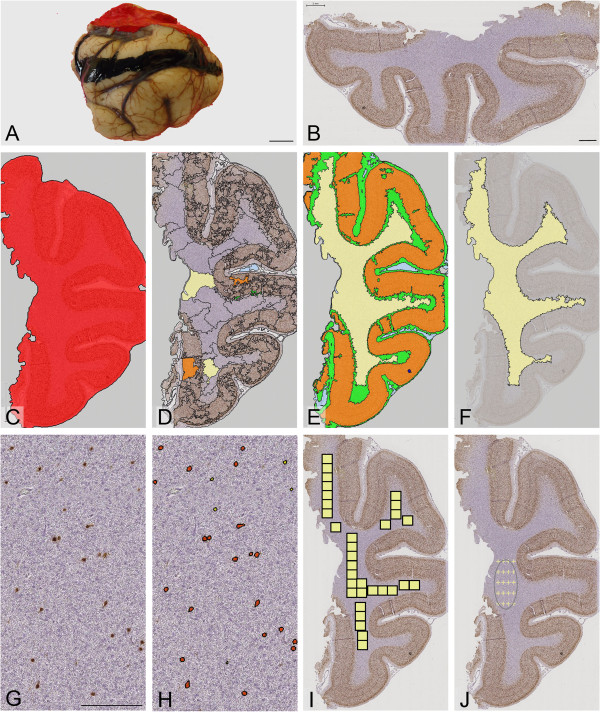
**Quantification of NeuN immunopositive neurons in the white matter. (A)** A surgically-resected temporal lobe from a patient with epilepsy who had undergone standard temporal lobectomy. **(B)** A 7μm-thick section was cut, immunolabelled using the neuronal marker, NeuN, and scanned using the whole slide scanner (LEICA). Using WSA, the white matter was selected automatically or manually on digitised images of NeuN immunopositive sections. In WSA automated, the programme identified the white matter by following a series of algorithms such as tissue and background separation **(C)**, and showed the training step, whereby the user selected ‘segments’ that were representative of the cortex (orange) or white matter (yellow; **D**), and the programme learned to identify other ‘patches’ of similar features **(E). (F)** In WSA manual, the user interactively outlined the white matter. After ROI selection, NeuN immunopositive cells in the white matter **(G)** of all cases were quantified by WSA **(H). (I)** In SA, images (yellow) were manually acquired by the user and then fed into a semi-quantitative analysis programme for quantification. **(J)** For stereology, a small area in the deep white matter was selected by the user and the programme moved through each point within the selection to allow the user to count NeuN immunopositive cells at 63× magnification. Scale bar = 1 cm **(A)**, 2 mm **(B-F, I-J)**, 200 μm **(G-H)**.

For WSA, sections were initially scanned on a LEICA SCN400F digital slide scanner (LEICA Microsystems, Wetzlar, Germany). Scanned images were processed into a Pyramid TIFF file, stored on a fileserver and viewed and managed on the SlidePath Digital Image Hub (LEICA). Analysis was carried out with Definiens Tissue Studio 3.6 (Definiens AG Munich, Germany). Prior to the study, a set of image analysis algorithms (“solutions”) with specific parameters were tested and optimised on Definiens Tissue Studio in a pilot study. The optimised parameters were applied as follows (Figure 1): First, tissue was selected from the background of each scanned image by setting homogeneity to 2.1 units, brightness to 200 units, and tissue minimum size to 450 μm^2^. By setting the initialisation magnification to 0.4 units, each section was then segmented into geographical regions based on the distribution of NeuN.

The images of 142 epilepsy and control cases were categorised into groups or ‘classes’ with similar intensity of haematoxylin staining and NeuN immunopositive labelling so the same algorithm could be applied to all sections of the same class; this would assist and speed up the user-training process. During the user-training process in WSA with automated ROI selection, the researcher ‘trained’ the program to recognise the cerebral cortex, grey/white matter border, white matter and empty spaces by selecting a few segments of well-defined areas within each region from two scanned images within each class. To optimise the area of white matter, filtering algorithms were applied to the grey/white matter boundary where the interface between compartments was blurred. Then the white matter was classified using the ‘nearest neighbour’ option, and relative area of less than 0.1 unit. These parameters/algorithms were then applied to all images in the same class.

For WSA with manual ROI selection, the researcher outlined the white matter of each image using a stylus pen on a touch-sensitive computer screen. This boundary was drawn at a set distance of 0.5 mm from the cortical margin, to exclude layer VI neurons. After either manual or automated ROI selection, WSA identified and counted NeuN immunopositive cells if labelled cells had an intensity of 3.1 units. All counts were expressed as cells per mm^2^ or mm^3^ adjusted by Abercrombie’s correction factor to correct for section thickness and mean cell size [28]. Actual section thickness was measured on the Zeiss Axio Imager Z2 microscope at 63× magnification, and the cell size of NeuN immunopositive labelled cells was measured using WSA. WSA also gave the percentage of NeuN immunopositive cells that were small (area of <126.36 μm^2^), medium (126.36 – 370 μm^2^) or large (>370 μm^2^).

For SA, 15 images of the white matter were pre-acquired from each scanned image at 10× magnification. NeuN immunopositive cells in each image were then automatically selected by thresholding based on the intensities in red, green, and blue channels.

49 epilepsy cases and one control were quantified using stereological cell counting methods employed in our previous published studies [11, 29, 30]. The region of interest in the deep white matter of each section was first outlined at 2.5× objective on a the Zeiss Axioskop microscope linked to a stereological software (Histometrix, Kinetic Imaging, Nottingham, UK). All NeuN immunopositive cells were counted with a 63× objective using the optical dissector with uniform random field sampling, and a sampling fraction of between 25–100% to minimise coefficient of error to 0.1 unit or less where possible.

Nonparametric Kruskal-Wallis and post-hoc comparisons, Mann–Whitney, Spearman correlation, and intraclass correlation coefficient tests were performed using SPSS 20 (IBM, USA). Correlations were conducted between density of WMN and age, gender, length of seizure history, history and frequency of simple partial seizures, generalised seizures, secondary generalised seizures, and various post-operative follow-ups (1^st^ to 18^th^). To test the reliability of each method, ten randomly-selected cases (two for stereology) were re-analysed. Bland and Altman plots were presented, and Pearson’s correlation and intra-class correlation coefficients using absolute agreement definition in a two-way mixed model, and single measure were calculated.

## Results

The quantitative techniques and results from WSA were compared with two other quantitative systems, SA, and stereology.

### Area of white matter quantified

The area of white matter sampled in 142 cases was significantly different between WSA automated and manual, SA and stereology (P < 0.000), but not between SA and stereology (P = 0.06). The range and average area sampled were the highest using WSA automated (246.1-6.4 mm^2^, 74.3 mm^2^), followed by WSA manual (178.8-3.4 mm^2^, 46.7 mm^2^), SA (7.1-0.4 mm^2^, 5.0 mm^2^) and stereology (11.9-1.2, 4.0 mm^2^) respectively; this is expected since the WSA allows the entire white matter to be sampled (Figure 1).

### ROI selection and identification of neurons

**Figure 2:**
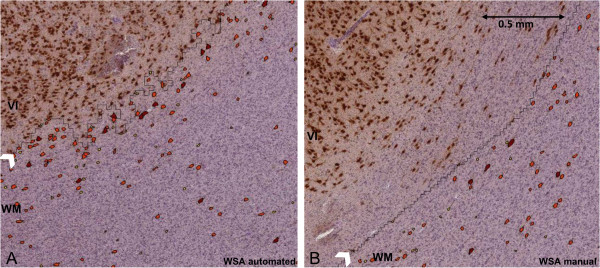
**Cell identification using WSA automated and manual. (A)** In some cases, WSA automated quantified NeuN immunopositive cells close to the grey/white matter demarcation (white arrow); some of these cells might be neurons in the cortical layer VI. **(B)** In contrast, because the user drew the white matter with a set distance away (0.5 mm) from the grey/white matter demarcation in WSA manual, we could be confident that the counted cells were in the white matter.

For the quantification of WMN, the ability of the quantitative systems to exclude neurons in the highly-populated cortical Layer VI at the cortical/white matter demarcation from the overall WMN counts is important. In stereology, SA and WSA manual, the user manually outlined a ROI that was clearly away from the cortex/white matter demarcation, so we could be confident that only neurons in the white matter were selected (Figure 2). In WSA automated, the program was trained to exclude areas in the cerebral cortex and the grey/white matter border, and only to quantify WMN within the white matter (Figure 2). When we reviewed the screenshot images of the counted cells for quality control purposes, we found that WSA automated correctly identified and quantified WMN in most cases; there were only a few occasions where WSA automated included some neurons from the lower limits of Layer VI.

NeuN immunopositive cells in all our cases were darkly labelled, and they were distinct from the background, so it was easy to train WSA and SA to accurately recognise and count these cells, and for the user to identify immunopositive cells in stereology using a 63× objective.

### Reliability of methods

**Figure 3:**
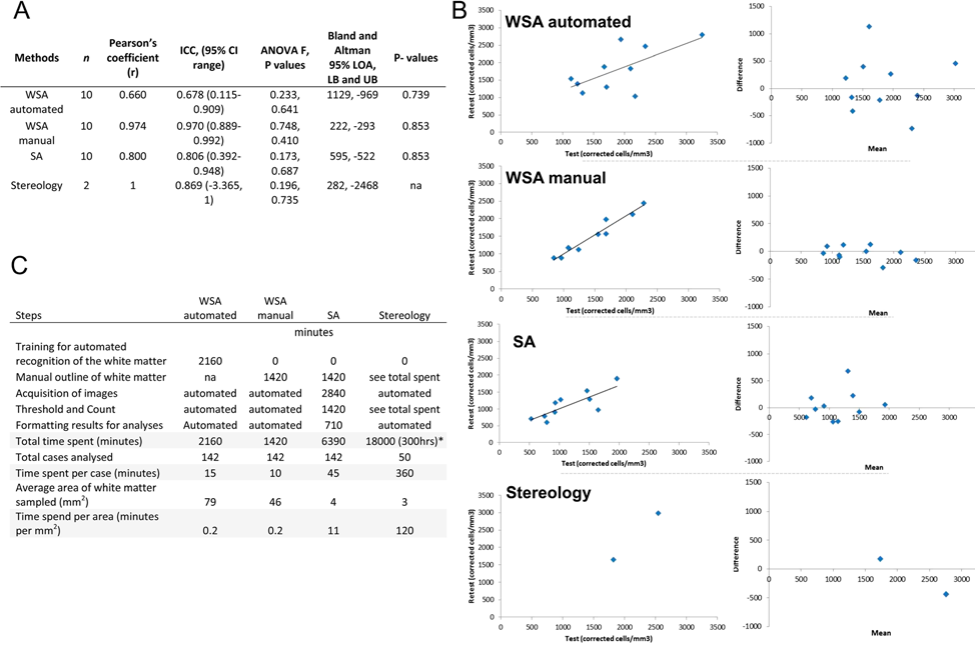
**Measure of reliability.** Ten cases (and two cases for stereology) were re-analysed using all methods. **(A)** Table showing Pearson’s correlation coefficient (*r*), intraclass correlation coefficients (ICC) with 95% confidence interval (CI), ANOVA, and non-parametric P values for all methods. The 95% level of agreement’s upper and lower boundary values (UB, LB) in Bland and Altman plots were also presented. **(B)** Graphs showing the correlation (left) and Bland & Altman plots (right) of WMN density values between test and retest using all methods. **(C)** Table showing the time spent on each method. *see Eriksson *et al*. [29].

Ten randomly selected cases in the series (and two cases for stereology) were re-analysed to determine the reliability of each method. No significant difference in density values was observed between test and re-test (P > 0.05) and the Pearson correlation coefficient (*r*) and the intraclass correlation coefficient (ICC), which are common measures of reliability [31], were above 0.6 for all methods (Figure 3A). Past studies indicate that the closer ICC and *r* are to one, the more reliable the method [31]. In our study, WSA manual had a Pearson’s correlation and an ICC value closest to one, suggesting that this method was the most reliable compared to other methods (Figure 3A-B). Bland and Altman plots also support these findings by showing good agreement with differences in most cases between test and retest values lying near zero (Figure 3B).

### Time consumption and ease of use

It took an average of six hours per case to quantify WMN using stereology [29] compared to 45 minutes per case using SA, 15 and ten minutes per case using WSA automated and manual respectively (Figure 3C). We found that WSA and SA were easier to use than stereology, with fewer steps involved.

### Density of WMN

**Figure 4:**
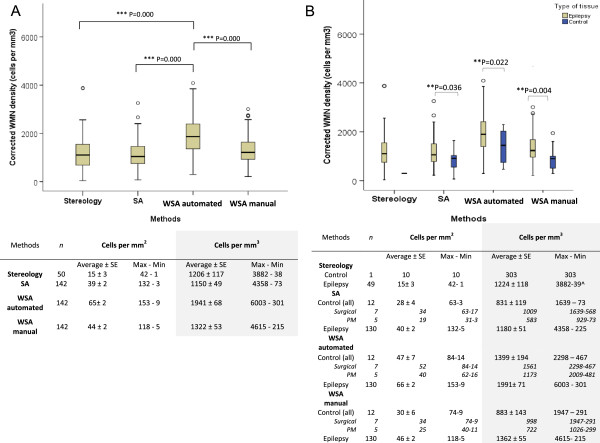
**Density of WMN. (A)** Boxplot showing quantitative results of all cases using WSA automated, WSA manual, SA and stereology. Horizontal line within box = median; top of the box = 75th percentile; bottom of box = 25th percentile; whiskers = 1.5x height of the box or maximum/minimum values. Tabulated results were expressed as cells/mm^2^ or cells/mm^3^ after Abercrombie’s correction ± standard error of means (SE). **(B)** Boxplot showing significantly higher densities of WMN in epilepsy cases compared to controls using all methods. ^The methodology in stereology was a 3D cell counting method, so correction factors were not applied.

The WMN density of all cases, corrected with the Abercrombie principle, was significantly different between WSA automated and WSA manual, SA, and stereology (P < 0.000; Figure 4A), but not between WSA manual and SA (P = 0.07), WSA manual and stereology (P = 0.130), or SA and stereology (P = 0.880). For epilepsy cases, the corrected WMN density was significantly different between all quantitative methods (P < 0.000), but not between methods when applied to controls (P = 0.269). The lack of differences between methods for control samples may be due to the small number of controls analysed, or because methods were less dissimilar if there were fewer WMN in the ROI. Subsequent post-hoc comparisons showed a significant difference in the corrected WMN density in epilepsy cases between WSA automated and WSA manual, SA, and stereology (P < 0.000) but not between WSA manual and SA (P = 0.073), WSA manual and stereology (P = 0.834), or SA and stereology (P = 0.706). The percentages of small, medium or large NeuN immunopositive cells in epilepsy cases were not significantly different between WSA automated and manual (P > 0.05).

### Comparison of WMN density between cases with epilepsy and controls

Quantitation revealed a significantly higher corrected density of WMN in up to 130 epilepsy cases compared to up to 12 controls using WSA automated (P = 0.022; Figure 4B), WSA manual (P = 0.004) and SA (P = 0.036). Only one control case was analysed using stereology and statistical analysis was hence not performed. The maximum WMN density for epilepsy cases in each method were two or three-fold higher than the maximum WMN density for controls. The corrected density of WMN in control and epilepsy cases did overlap but no control case had over 2298 cells/mm^3^ using WSA automated. Our controls included five post mortem, normal cases, and seven surgical, non-epilepsy cases. Surgical tissue from neurologically normal patients is by definition not available. Therefore, we used as controls histologically normal, surgically resected tissue from non-epileptic patients with traumatic brain injury or tumours. No significant difference in WMN density was observed between these groups of controls using WSA automated and manual, and SA. The corrected WMN density was significantly different between epilepsy cases and post mortem controls (P < 0.05), but not against surgical non-epilepsy controls using WSA automated and manual selections, and SA. We cannot exclude that subtle oedema or mild gliosis in our surgical non-epilepsy controls may have influenced the density and location of WMN neurons in these surgical controls.

The area of white matter sampled, thickness, and average cell diameter were not significantly different between epilepsy cases and controls using any of the methods (P > 0.05). The percentages of small, medium, and large NeuN immunopositive cells and their average intensity measured by WSA automated were not significantly different between epilepsy cases and post mortem and surgical controls (Additional file 4: Table S4).

No significant correlation between the corrected density of WMN in epilepsy cases and clinical parameters was observed after Bonferroni correction for multiple comparison was applied at P < 0.002.

## Discussion

This is the first study that has investigated and compared the reliability, accuracy, the ease of use and time consumption of three quantitative systems, WSA, SA, and stereology. We found that WSA sampled larger regions, performed faster analysis, and provided the most reproducible results compared to other systems. Using WSA, we found that a higher density of WMN in the 130 epilepsy cases compared to controls, but the density of WMN in epilepsy cases did not correlate with clinical parameters relating to seizure frequency or surgical outcome.

With the introduction of many different systems for quantitative histology, it is important that systems are rigorously tested for both reliability and cost-effectiveness, especially if results are to be used to assist in clinical diagnosis. Consequently, we compared the quantitative findings from three systems, employing three different counting approaches; stereology, SA and WSA. Three-dimension stereology or design-based stereology has been long considered as the most accurate method to quantify neurons in an ‘optically-defined volume of tissue’ [27]. Stereology avoids the need to correct for variation in section thickness and cell size of each case as established in the Abercrombie principle [28, 32], with confident, unbiased live counting of neurons at high magnification using 63 or 100× objectives. However, stereology is time-consuming, taking approximately six hours per case [29], compared to ten to 15 minutes per case with a maximally automated system such as WSA. Stereology is, therefore, impractical in a diagnostic laboratory. Findings from this study suggest that automated systems such as WSA may be a good alternative to stereology, considering that no significant difference in WMN density values was observed between stereology and WSA manual, and the results obtained from WSA manual were reproducible and reliable as evident in the intra-rater reliability coefficient tests. Furthermore, the WSA system is superior over stereology: WSA has the power to examine a large area and it avoids user-selected bias of only small areas of interest (for SA and stereology) which may not be representative of the whole sample; it can also quantify sections rapidly, thus allowing for more cases and additional morphometric data to be examined without adding extra time to the count data (whereas in stereology, the use of the optical dissector method to measure the size of each cell drastically increases the analysis time). Together, these advantages support the use of WSA in quantitative histology.

**Figure 5:**
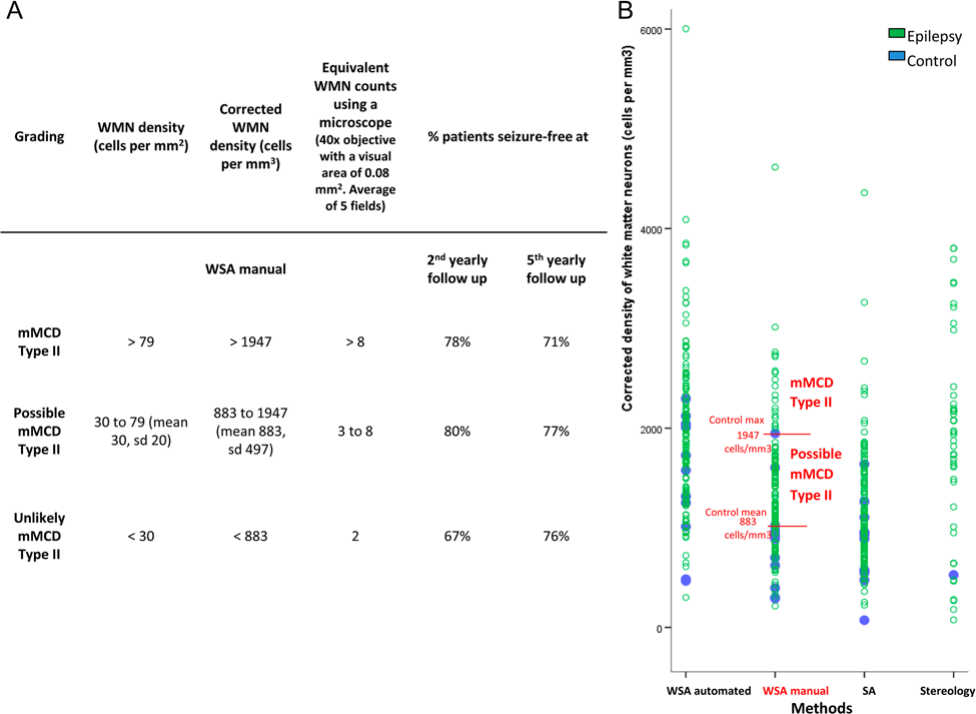
**Proposed criteria for the classification of mMCD II in epilepsy. (A)** This may be used as practical guidelines for neuropathologists to diagnose mMCD II in the temporal cortical resections from patients with mTLE and hippocampal sclerosis. Values are based on the present study which have used cortical resections (inclusive of the superior, middle, and inferior temporal gyrus that were 1–1.5 cm from pole), and immunohistochemistry using anti-NeuN (Millipore MAB377, 1:100, overnight). **(B)** In this study, WMN density values obtained using WSA manual, whereby the area of interest was outlined a set distance (0.5 mm) from the grey-white matter boundary, was regarded as the gold standard in the evaluation of neuronal counts. A graphical representation of our proposed numerical thresholds is presented here. Each point represents one case. WSA = whole slide imaging with automated analysis, control max = the highest WMN density value from controls, control mean = mean WMN density of controls, mMCD = mild Malformation of Cortical Development, SA = semi-automated analysis, sd = standard deviation, WMN = white matter neurons.

Establishing an agreed numerical threshold based on the density of WMN to aid the diagnosis of mMCD II in humans is a fundamental aim for neuropathologists working in epilepsy surgical centres [2]. This information is lacking, however, mainly because previous studies have used different stains, quantitative methods, and type of specimens (see Additional file 1: Table S1; [33]). Here, using WSA on a large series of cases with uniform pathology, surgical procedure, standardised tissue processing, staining and selection of anatomical compartment, thus forming an optimal group of cases to address this research question, we found that all techniques showed a significant increase of WMN density in the epilepsy cases compared to normal post mortem controls, which was consistent with previous quantitative studies (see Additional file 1: Table S1). We found that, while the range of WMN density values from controls and epilepsy cases did overlap (consistent with [9, 11, 34, 35]), we did not observe any control case with a corrected WMN density of over 2298 cells/mm^3^ (WSA with automated selection) regardless of which quantitative method was used. Previously, Blümcke *et al*. suggested that a WMN density excess of 20 cells/mm^2^ might be used to diagnosis mMCD II [33], but as we found that some of our normal post mortem cases had over 20 cells/mm^2^ regardless of quantitative methods, we propose a scale for the certainty of the diagnosis based on results obtained using WSA with manual selection (Figure 5). For the quantitation of WMN, WSA with manual selection is faster and more consistent and reliable in identifying neurons in the white matter compared to the complete automated selection; in the manual selection, the user drew a selection that was a set distance from the grey/white matter demarcation, therefore excluding the cortical layer VI.

The cause of the increased WMN density in epilepsy is unknown. The failure of neuronal migration, and/or the elimination or de novo generation of WMN, as either a cause or effect of seizures, have all been postulated but remain to be proven. In regards to their influence on epileptogenesis, previous studies on the functional activity of WMN (normal or pathological) are limited and conflicting [5, 18, 36–40]. Three studies have reported that WMN density may have a predictive effect on seizure outcome following surgical resection which would support a role of increased WMN density in seizure networks in the temporal lobe [9, 11, 14], but this finding was not observed in other similar studies [10].

## Conclusion

This study showed that WSA, a modern high-throughput, automated analysis is more efficient in selecting larger regions and cells of interest, and in processing cell counts and morphometric data compared to the other systems. These obvious advantages of whole slide scanning have to be balanced with the practicalities and expense in that not every neuropathology laboratory currently has access to slide scanners and a whole slide image analysis software solution. Furthermore, users should be reminded that both WSA and SA rely on user-defined size and/or thresholding parameters to identify cells of interest, so the accuracy of quantification will ultimately be dependent on the users’ settings and the quality of the acquired images/scans. Therefore, quality assurance by trained personnel after defining the parameters and repeated counts will be important. Nevertheless, the high-throughput data generated with the WSA systems can be used as a ‘gold standard’ to provide bench-mark criteria (Figure 5) to translate into diagnostic practice using a conventional microscope for the future assessment of mild MCDs.

## Electronic supplementary material

Additional file 1: Table S1: Previous studies that have investigated WMN in adult patients with epilepsy. H&E, Haematoxylin & Eosin; LFB-PAS, Luxol Fast Blue- Periodic Acid Schiff; Map2, microtubule associated protein 2; NeuN, neuronal nuclei antigen; ROI, region of interest. (DOCX 23 KB)

Additional file 2: Table S2: The US Food and Drug Administration has approved the digital whole slide imaging hardware and analytic algorithms of the above companies for the diagnostic evaluation or screening of HER2, PR, ER, Ki67 and/or p53 (Digital Pathology Associations). (DOCX 20 KB)

Additional file 3: Table S3: Summary of clinical details from patients with epilepsy and non-epilepsy surgical controls. *Surgical outcome was classified according to International League against epilepsy (ILAE, Wieser et al. [41]) where 1 = completely seizure free and no aura; 2 = only auras and no other seizures; 3 = one to three seizures days per year with/out auras; 4 = four seizures days per year to 50% reduction of baseline seizure days with/out auras; 5 = less than 50% reduction of baseline seizure days to 100% increase of baseline seizure days with/out auras; 6 = more than 100% increase of baseline seizure days with/out auras; na = unknown. sd = standard deviation. (DOCX 21 KB)

Additional file 4: Table S4: Size and intensity of NeuN immunopositive cells measured using WSA automated. The percentage of NeuN immunopositive cells with an area of less than 126.36 μm2 (small), between 126.36-370 μm2 (medium) and over 370 μm2 (large) were not significantly different between epilepsy and control cases. (DOCX 19 KB)

## Abbreviations

WSA: Whole slide imaging with automated analysis
ICC: Intraclass correlation coefficient
mMCD: mild Malformation of Cortical Development
MRI: Magnetic resonance imaging
NeuN: Neuronal nuclei
ROI: Region of interest
SA: Semi-automated analysis
WMN: White matter neurons.
