# Supplementary material for: High-throughput, automated quantification of white matter neurons in mild malformation of cortical development in epilepsy
Source: Acta Neuropathol Commun. 2014 Jun 13;2:72. doi: 10.1186/2051-5960-2-72 (PMC4229809; doi:10.1186/2051-5960-2-72)
Supplement: Supplementary file 1 — Additional file 1: Table S1: Previous studies that have investigated WMN in adult patients with epilepsy. H&E, Haematoxylin & Eosin; LFB-PAS, Luxol Fast Blue- Periodic Acid Schiff; Map2, microtubule associated protein 2; NeuN, neuronal nuclei antigen; ROI, region of interest. (DOCX 23 KB) [file 40478_2014_139_MOESM1_ESM.docx]

| **Published studies** | **Samples and regions** | **Findings** |
| --- | --- | --- |
| Meencke and Janz, 1984 | 8 cases with primary generalised epilepsy | Qualitative assessment with report of increased nerve cells in the white matter. |
| Hardiman et al., 1988 | 50 surgical epilepsy and 33 normal autopsy cases. ROI within temporal deep white matter. | 42% of cases with epilepsy had a density of over 8 neurons per 2 mm^2^ the white matter, while no neurons were observed in the deep white matter of controls. The presence of neuronal ectopia had a favourable clinical outcome following surgery |
| Rojiani et al., 1996 | 20 normal autopsy cases. ROI within the frontal, occipital, temporal deep white matter. | Neurons were counted at 40x magnification by an assessor on LFB-PAS stained sections. Temporal (2.35 neurons/mm^2^), Frontal (0.38) Occipital (0.35) |
| Emery et al., 1997 | 22 surgical epilepsy and 22 normal autopsy cases. ROI within Temporal deep white matter (2-3 mm deep to the cortex) | Only large neurons with nuclei over 12 um in diameter were counted by an assessor on LFB-PAS (epilepsy 4.11 neurones/mm^2^, control 2.35).  Large Map2 neurons over 15um diameter were also counted (Epilepsy 4.08 neurons/mm^2^, control 1.68 neurons/mm^2^) |
| Kasper et al., 1999 | 47 surgical TLE (50% with HS) and 29 normal autopsy cases. Temporal. | The numbers of neurons on H+E/ Nissl stained slides were counted per HPF (x 400 magnification) and categorized as 0, 1-2, 3-10 or more than 10. 23% of cases with epilepsy had > 10 neurons/HPF in the deep white matter, which was not observed in any controls. Increased white matter neurons had a worse postoperative outcome in epilepsy cases. |
| Bothwell et al 2001 | 8 surgical epilepsy and 8 normal autopsy cases. ROI within Brodmann area 38 (anterior pole of the temporal pole) | Design-based stereology on Nissl stained sections.  Epilepsy 1950-440 neurons/mm^3^, (average 1160 mm^3^).  Control 1350-350 neurons/mm^3^, (750) |
| Thom et al., 2001 | 31 surgical epilepsy, and 15 controls (4 surgical, 11 autopsy). Temporal. | Design-based stereology on Nissl and NeuN labelled sections.  Nissl (Epilepsy 1751-440/mm^3^ (average, 1010) , Control not available)  NeuN (Epilepsy 3448-1212/mm^3^ (average, 2164), Control 2990-620/mm^3^, (1660)) |
| Eriksson et al., 2006 | 10 surgical epilepsy cases. Deep white matter of the middle temporal gyrus (ROI ranged from 3.96-10.3 mm^2^) | Design-based stereology on NeuN labelled sections. Epilepsy (4910-2120/mm^3^ (average, 2873), no controls)  2D automated quantitation on NeuN labelled sections. Epilepsy (47.1- 13.2 neurons/mm^2^, No control group |
| Judas et al., 2010 | 13 fetal, 6 infants/ children and 6 adult normal autopsy cases. Whole brain | Qualitative assessment of white matter neurons. |
| Garbelli et al., 2011 | 13 surgical TLE with HS cases. | Line profiles showing the Intensity of NeuN-labelling across the grey white matter |
| Lockwood-Estrin et al., 2011 | 24 surgical epilepsy cases. Deep white matter of the middle temporal gyrus (ROI ranged from 250-500 at 63x field of view) | Design-based stereology on NeuN labelled sections. Epilepsy (7.28-1.65x 10^-6^/µm^3^ (average, 3.73), no controls) |
| Mulhebner et al., 2012 | 52 surgical samples from patients with FCD. ROI within the deep white matter (500 µm from grey-white matter demarcation) of various brain regions. Sample area of 2.25 mm^2^.  90 controls (24 autopsy, and 12 surgical cases) | Map2 signal intensity suggested that there was an increase in the number of neurons in the deep white matter of all epilepsy specimens compared to controls. |

**Additional file 1: Table S1**
