# Supplementary material for: High-throughput, automated quantification of white matter neurons in mild malformation of cortical development in epilepsy
Source: Acta Neuropathol Commun. 2014 Jun 13;2:72. doi: 10.1186/2051-5960-2-72 (PMC4229809; doi:10.1186/2051-5960-2-72)
Supplement: Supplementary file 2 — Additional file 2: Table S2: The US Food and Drug Administration has approved the digital whole slide imaging hardware and analytic algorithms of the above companies for the diagnostic evaluation or screening of HER2, PR, ER, Ki67 and/or p53 (Digital Pathology Associations). (DOCX 20 KB) [file 40478_2014_139_MOESM2_ESM.docx]

| **Company** | **Year** | **Tissue- Stains** | **IHC reagents** | **Application** |
| --- | --- | --- | --- | --- |
| Leica Microsystems, Germany Aperio | 2007 | Breast- HER2/neu | DAKO | Image Analysis |
| Leica (Aperio) | 2008 | Breast- ER/ PR | DAKO | Image Analysis |
| Leica (Aperio) | 2009 | Breast- HER2/neu | DAKO | Tunable image analysis |
| Leica (Applied Imaging) | 2004 | Breast- ER/ PR, HER2/neu | DAKO | Image Analysis |
| Zeiss (Clarient/ Chromavision) | 2003 | Breast- HER2/neu | DAKO | Image Analysis |
| Zeiss (Clarient/ Chromavision) | 2004 | Breast- ER/ PR | DAKO | Image Analysis |
| Roche/ Ventana Medical Systems, US (Tripath) | 2005 | Breast- ER/PR, HER2/neu | Ventana | Image Analysis |
| Roche/ Ventana (Tripath) | 2006 | Breast- Ki-67, p53 | Ventana | Image Analysis |
| Roche/ Ventana (Bioimagene) | 2007, 2009 | Breast- HER2/neu | DAKO | Image Analysis |
| Roche/ Ventana (Bioimagene) | 2010 | Breast p53/ Ki-67 | DAKO | Image Analysis |
| Ventana | 2011 | Breast- HER2 (4B5) | Ventana | Image Analysis |

**Additional file 2: Table S2**
