# Supplementary material for: High-throughput, automated quantification of white matter neurons in mild malformation of cortical development in epilepsy
Source: Acta Neuropathol Commun. 2014 Jun 13;2:72. doi: 10.1186/2051-5960-2-72 (PMC4229809; doi:10.1186/2051-5960-2-72)
Supplement: Supplementary file 3 — Additional file 3: Table S3: Summary of clinical details from patients with epilepsy and non-epilepsy surgical controls. *Surgical outcome was classified according to International League against epilepsy (ILAE, Wieser et al. [41]) where 1 = completely seizure free and no aura; 2 = only auras and no other seizures; 3 = one to three seizures days per year with/out auras; 4 = four seizures days per year to 50% reduction of baseline seizure days with/out auras; 5 = less than 50% reduction of baseline seizure days to 100% increase of baseline seizure days with/out auras; 6 = more than 100% increase of baseline seizure days with/out auras; na = unknown. sd = standard deviation. (DOCX 21 KB) [file 40478_2014_139_MOESM3_ESM.docx]

Epilepsy cases (n=130)

| Parameters | Description |
| --- | --- |
| Gender | Female (n=68), Male (n=62) |
| Average seizure history (sd) | 27 years (11) |
| Average age of epilepsy onset (sd) | 10 years (8) |
| Average age at surgery (sd) | 37 years (10) |
| Hemisphere of lesion | Left (n=60), Right (n=70) |
| History of status epilepticus | Unknown (n=4), Yes (n=20), No (n=106) |
| History of simple partial seizures | Unknown (n=11), Yes (n=73, average 23 seizures/month), No (n=46) |
| History of complex partial seizures | Unknown (n=1), Yes (n=124, average 12 seizures/month), No (n=5) |
| History of secondarily generalised seizures | Yes (n=97, average 1 seizure/month), No (n=33) |
| Surgical outcome at post-operational yearly follow-ups* | 1^st^:1 (n=80), 2(n=17), 3(n=14), 4(n=14), 5(n=2), na(n=1) |
|  | 2^nd^: 1 (n=79), 2(n=17), 3(n=11), 4(n=15), 5(n=3), na(n=5) |
|  | 3^rd^: 1 (n=72), 2(n=10), 3(n=19), 4(n=14), 5(n=6), na(n=9) |
|  | 4^th^: 1 (n=67), 2(n=10), 3(n=13), 4(n=14), 5(n=5), na(n=21) |
|  | 5^th^: 1 (n=65), 2(n=12), 3(n=6), 4(n=17), 5(n=2), na(n=28) |
|  | 6^th^: 1 (n=63), 2(n=9), 3(n=8), 4(n=12), 5(n=2), na(n=36) |
|  | 7^th^: 1 (n=46), 2(n=9), 3(n=5), 4(n=8), 5(n=1), na(n=61) |
|  | 8^th^: 1 (n=41), 2(n=7), 3(n=1), 4(n=6), na(n=75) |
|  | 9^th^: 1 (n=26), 2(n=7), 3(n=2), 4(n=5), na(n=90) |
|  | 10^th^: 1 (n=26), 2(n=6), 3(n=1), 4(n=4), 5(n=1), na(n=97) |
|  | 11^th^: 1 (n=17), 2(n=5), 3(n=0), 4(n=4), 5(n=1), na(n=103) |
|  | 12^th^: 1 (n=15), 2(n=2), 3(n=2), 4(n=3), 5(n=1), na(n=109) |
|  | 13^th^: 1 (n=14), 2(n=3), 4(n=2), 5(n=109) |
|  | 14^th^: 1 (n=13), 2(n=2), 4(n=1), 5(n=2), na(n=112) |
|  | 15^th^: 1 (n=13), 2(n=3), 3(n=1), 5(n=120) |
|  | 16^th^: 1 (n=4), 2(n=1), na(n=116) |
|  | 17^th^: 1 (n=4), 2(n=1), na (n=125) |
|  | 18^th^: 1 (n=2), 2(n=2), na (n=121) |

Control: non-epilepsy surgical cases (n=7)

| Parameters | Description |
| --- | --- |
| Gender | Female (n=2), Male (n=5) |
| Average age at surgery (sd) | 39 years (12) |
| Reason for surgery (and underlying pathology) | Traumatic brain injury (n=3), Tumour (n=4: glioblastoma multiforme WHO grade IV (1), oligoastrocytoma WHO grade II, astroglia tumour (no further grading possible), mestastic carcinoma |

**Additional file 3: Table S3**
