# Supplementary material for: High-throughput, automated quantification of white matter neurons in mild malformation of cortical development in epilepsy
Source: Acta Neuropathol Commun. 2014 Jun 13;2:72. doi: 10.1186/2051-5960-2-72 (PMC4229809; doi:10.1186/2051-5960-2-72)
Supplement: Supplementary file 4 — Additional file 4: Table S4: Size and intensity of NeuN immunopositive cells measured using WSA automated. The percentage of NeuN immunopositive cells with an area of less than 126.36 μm2 (small), between 126.36-370 μm2 (medium) and over 370 μm2 (large) were not significantly different between epilepsy and control cases. (DOCX 19 KB) [file 40478_2014_139_MOESM4_ESM.docx]

| **WSA automated** | **n** | **Average % small NeuN+ cells** | **Average % medium-sized NeuN+ cells** | **Average % large NeuN+ cells** | **Average intensity of NeuN+ cells** |
| --- | --- | --- | --- | --- | --- |
| Control | 12 | 44 | 48 | 11 | 0.76 |
| Epilepsy | 130 | 44 (P=0.823) | 47 (P=0.415) | 8 (P=0.886) | 0.77 |

**Additional file 4: Table S4**
